# Glutamate 73 Promotes Anti-arrhythmic Effects of Voltage-Dependent Anion Channel Through Regulation of Mitochondrial Ca^2+^ Uptake

**DOI:** 10.3389/fphys.2021.724828

**Published:** 2021-08-18

**Authors:** Hirohito Shimizu, Simon Huber, Adam D. Langenbacher, Lauren Crisman, Jie Huang, Kevin Wang, Fabiola Wilting, Thomas Gudermann, Johann Schredelseker, Jau-Nian Chen

**Affiliations:** ^1^Department of Molecular, Cell and Developmental Biology, University of California, Los Angeles, Los Angeles, CA, United States; ^2^Walther Straub Institute of Pharmacology and Toxicology, Faculty of Medicine, LMU Munich, Munich, Germany; ^3^German Centre for Cardiovascular Research (DZHK), Partner Site Munich Heart Alliance, Munich, Germany

**Keywords:** mitochondria, voltage-dependent anion channel, calcium, cardiac rhythmicity, zebrafish

## Abstract

Mitochondria critically regulate a range of cellular processes including bioenergetics, cellular metabolism, apoptosis, and cellular Ca^2+^ signaling. The voltage-dependent anion channel (VDAC) functions as a passageway for the exchange of ions, including Ca^2+^, across the outer mitochondrial membrane. In cardiomyocytes, genetic or pharmacological activation of isoform 2 of VDAC (VDAC2) effectively potentiates mitochondrial Ca^2+^ uptake and suppresses Ca^2+^ overload-induced arrhythmogenic events. However, molecular mechanisms by which VDAC2 controls mitochondrial Ca^2+^ transport and thereby influences cardiac rhythmicity remain elusive. Vertebrates express three highly homologous VDAC isoforms. Here, we used the zebrafish *tremblor/ncx1h* mutant to dissect the isoform-specific roles of VDAC proteins in Ca^2+^ handling. We found that overexpression of VDAC1 or VDAC2, but not VDAC3, suppresses the fibrillation-like phenotype in zebrafish *tremblor/ncx1h* mutants. A chimeric approach showed that moieties in the N-terminal half of VDAC are responsible for their divergent functions in cardiac biology. Phylogenetic analysis further revealed that a glutamate at position 73, which was previously described to be an important regulator of VDAC function, is sevolutionarily conserved in VDAC1 and VDAC2, whereas a glutamine occupies position 73 (Q73) of VDAC3. To investigate whether E73/Q73 determines VDAC isoform-specific anti-arrhythmic effect, we mutated E73 to Q in VDAC2 (VDAC2^E73Q^) and Q73 to E in VDAC3 (VDAC3^Q73E^). Interestingly, VDAC2^E73Q^ failed to restore rhythmic cardiac contractions in *ncx1* deficient hearts, while the Q73E conversion induced a gain of function in VDAC3. In HL-1 cardiomyocytes, VDAC2 knockdown diminished the transfer of Ca^2+^ from the SR into mitochondria and overexpression of VDAC2 or VDAC3^Q73E^ restored SR-mitochondrial Ca^2+^ transfer in VDAC2 deficient HL-1 cells, whereas this rescue effect was absent for VDAC3 and drastically compromised for VDAC2^E73Q^. Collectively, our findings demonstrate a critical role for the evolutionary conserved E73 in determining the anti-arrhythmic effect of VDAC isoforms through modulating Ca^2+^ cross-talk between the SR and mitochondria in cardiomyocytes.

## Introduction

Mitochondria govern a variety of cellular processes including bioenergetics, metabolism, reactive oxygen species production, cell survival, and Ca^2+^ homeostasis. In particular, a tight cross-talk between mitochondria and the endoplasmic reticulum (ER) or sarcoplasmic reticulum (SR) was associated with important physiological and pathophysiological processes (Rizzuto et al., 1998; Gomez et al., 2016; Paillusson et al., 2016; Dia et al., 2020). In the heart, the close proximity of the SR and mitochondria facilitates Ca^2+^ cross-talk between these cellular organelles and provides a mechanism for the regulation of cardiac Ca^2+^ handling and adaptation to higher workload. This SR-mitochondria cross-talk depends on an array of inner and outer mitochondrial membrane channels, including the highly abundant voltage-dependent anion channels (VDACs) in the outer mitochondrial membrane, which critically regulate transport of ions, substrates, and metabolites across the outer mitochondrial membrane. VDAC dysfunction has previously been linked to pathophysiological outcomes (Feng et al., 2019; Santin et al., 2020), but the roles of VDAC proteins in cardiac function and disease are still poorly understood.

The vertebrate VDAC family consists of three paralogs, VDAC1, VDAC2, and VDAC3. While these proteins are highly similar in their sequences, structures, and biophysical properties, different biological functions have been noted among these isoforms (Naghdi and Hajnóczky, 2016; Caterino et al., 2017). In particular, there is a distinct requirement for VDAC2 during mammalian embryogenesis: Mice without functional VDAC1 or VDAC3 can survive to adulthood (Anflous et al., 2001; Sampson et al., 2001), but VDAC2 deficiency leads to embryonic lethality (Cheng et al., 2003). Furthermore, conditional ablation of VDAC2 specifically in cardiomyocytes causes cardiomyopathy suggesting an essential role for VDAC2 in maintaining the physiological function of the heart (Raghavan et al., 2012).

In cardiomyocytes, VDAC2 serves as a gatekeeper for transferring Ca^2+^ into mitochondria under both normal physiological and stressed conditions (Rosencrans et al., 2021; Sander et al., 2021). For example, knockdown of VDAC2 significantly extended Ca^2+^ sparks, supporting a physiological role for VDAC2 in Ca^2+^ cycling (Subedi et al., 2011; Min et al., 2012). Our laboratories previously showed that enhancing mitochondrial Ca^2+^ uptake with efsevin, a gating modifier of VDAC2 (Wilting et al., 2020), can suppress arrhythmogenesis in cardiomyocytes (Shimizu et al., 2015; Schweitzer et al., 2017). Efsevin efficiently restored rhythmic cardiac contractions in zebrafish *tremblor/ncx1h* mutant embryos, which are characterized by cardiomyocyte Ca^2+^ overload and chaotic cardiac contractions (Ebert et al., 2005; Langenbacher et al., 2005; Shimizu et al., 2015) and suppressed erratic, diastolic Ca^2+^ events in both a murine model for human catecholaminergic polymorphic ventricular tachycardia (CPVT) and in iPSC-derived cardiomyocytes from a CPVT patient (Schweitzer et al., 2017).

Overexpression of VDAC2 allows *tremblor/ncx1h* mutant zebrafish hearts to establish coordinated cardiac contractions and to preserve the integrity of myofibrils (Shimizu et al., 2015, 2017), indicating that *tremblor/ncx1h* can serve as an animal model for the study of cardiac Ca^2+^ regulation by VDAC. In this study, we investigate the potential of the three VDAC isoforms to regulate Ca^2+^ signaling in the heart, with the aim of identifying molecular moieties which confer isoform specificity. We show that overexpression of VDAC1, but not VDAC3, restored regular contractions in *tremblor/ncx1h* mutants comparable to VDAC2. We further show that VDAC1 and VDAC2 possess a glutamate at position 73 (E73), whereas VDAC3 contains a glutamine (Q73), a residue that was previously reported to be involved in VDAC regulation. Strikingly, substitution of VDAC3 Q73 for a glutamate confers a gain of function ability to restore rhythmic cardiac contractions in *tremblor/ncx1h* mutants while the converse exchange in VDAC2 abolishes its phenotype rescue ability. Finally, using cellular assays, we show that the glutamate at position 73 of VDAC proteins is essential for mediating mitochondrial Ca^2+^ uptake.

## Materials and Methods

### Phylogenetic Analysis

Protein sequences used for phylogenetic analyses were downloaded from the NCBI database.^1^ A full list of the NCBI accession IDs for each sequence used is found in Supplementary File 1. For analysis of vertebrate VDACs, full-length VDAC protein sequences were aligned using the MUSCLE algorithm in the MEGA7 application (Kumar et al., 2016), producing a 284 position alignment. For expanded analysis of vertebrate and non-vertebrate VDACs, full-length protein sequences were aligned using the ClustalW algorithm in MEGA7, producing a 397 position alignment. Phylogenetic analyses were performed with the software RAxML using a maximum likelihood method, the JTT substitution matrix, and empirical frequencies (Stamatakis, 2014). RAxML software was accessed using the CIPRES Science Gateway (Miller et al., 2010) and trees were visualized using the Interactive Tree of Life website (Letunic and Bork, 2007).

### Pairwise Comparison of VDAC Proteins

Human and zebrafish VDAC protein sequences were downloaded from the NCBI database and their percent identity was determined using a standard protein BLAST (blastp). Pairwise comparisons were visualized in R v3.6.3 using the ggcorrplot package.

### Cloning

Plasmids containing the full-length zebrafish VDAC1, 2, and 3 cDNA were purchased from Open Biosystems and cloned into pCS2+ or pCS2+3xFLAG plasmid for mRNA synthesis. Point mutations VDAC2^E73Q^ and VDAC3^Q73E^ were inserted by SOE-PCR.

For HeLa cell transfections, coding regions of VDAC genes were cloned into the pCS2+ vector together with the NLS-EGFP fragments and the viral T2A sequence. The T2A sequence enables bicistronic expression of VDAC proteins and NLS-EGFP for the identification of VDAC-overexpressing cells.

Plasmids for the generation of transgenic lines were generated using the Tol2Kit (Kwan et al., 2007). Plasmids shLenti2.4G-mVDAC2 and shLenti2.4G-Ctrl for VDAC2 knockdown in HL-1 cells were generously provided by Dr. Yeon Soo Kim from Inje University, Gimhae, South Korea. Plasmids pCCLc-CMV, pCMVΔ8.91, and pCAGGS-VSV-G for lentivirus production were obtained as a gift from Dr. Donald Kohn, University of California Los Angeles, United States. For production of lentiviruses, the IRES-nlsEGFP element from p3E-IRES-nlsEGFPpA (Kwan et al., 2007) was fused to VDAC elements by subcloning into pCS2+ constructs, before the entire VDAC-eGFP element was fused into pCCLc-CMV using the In-Fusion HD Cloning Kit (TaKaRa; Wilting et al., 2020).

### Zebrafish Husbandry, Generation of Transgenic Fish Lines, and Chemical Induction

Zebrafish were raised and maintained under standard laboratory conditions. The zebrafish *tremblor* (*tre^tc318d^*) mutant line (ZDB-ALT-980203-1756) was bred and maintained as previously described (Langenbacher et al., 2005). To induce transgene expression in *Tg(myl7:Gal4EcR-EGFP-UAS-vdac1-FLAG)*, *Tg(myl7:Gal4EcR-EGFP-UAS-vdac2-FLAG)*, and *Tg(myl7:Gal4EcR-EGFP-UAS-vdac3-FLAG),* 1 μM tebufenozide (TBF) was added to the embryo media at 1 day post-fertilization (dpf; Lu et al., 2017). Cardiac phenotypes were assessed at 2 dpf (Shimizu et al., 2015, 2017).

### Zebrafish Injections

mRNA was synthesized from pCS2+-constructs using the SP6 mMESSAGE mMACHINE kit (Life Technologies). mRNA was injected into one-cell stage embryos collected from crosses of *tre^tc318^* heterozygotes. Cardiac performance was analyzed by visual inspection of cardiac contractions on 1 dpf. and genotypes were confirmed at 2–3 dpf.

### Zebrafish Expression Profiling

Total RNA of embryos was isolated using Trizol Reagent (Life Technologies) and cDNA was synthesized using the iScript cDNA synthesis kit (Bio-Rad). Sequences for forward and reverse primers used for RT-PCR are provided in Supplementary File 2.

### *In-Situ* Hybridization

Whole-mount *in situ* hybridization was performed as previously described (Chen and Fishman, 1996) using VDAC1, 2, and 3 probes spanning the entire coding sequences. For riboprobe synthesis, plasmids were linearized and *in vitro* transcription was performed using the DIG RNA labeling kit (Roche).

### HeLa Ca^2+^ Uptake Assay

HeLa cells were transfected with expression plasmids using Lipofectamine2000 (Life Technologies). 24 h after transfection, cells were loaded with 5 μM Rhod2-AM (Life Technologies) in loading buffer (5 mM HEPES, 140 mM NaCl, 4 mM KCl, 1 mM MgCl_2_, 10 mM glucose, and pH adjusted to 7.4 with NaOH) for 1 h at 15°C followed by an additional 30 min incubation in wash buffer (10 mM HEPES, 140 mM TEA-Cl, 1 mM MgCl_2_, 2 mM Na-EGTA, 10 mM glucose, and pH adjusted to 7.4 with Trizma base) at 37°C to allow de-esterification of cytosolic AM esters. Prior to imaging, cells were permeabilized with 100 μM digitonin for 1 min at room temperature. After approximately 10 s of baseline recording, cells were exposed to an external Ca^2+^ pulse (final free Ca^2+^ concentration is calculated to be approximately 10 μM using WEBMAXC at https://somapp.ucdmc.ucdavis.edu/pharmacology/bers/maxchelator/webmaxc/webmaxcS.htm). Confocal images were recorded in internal buffer (5 mM K-EGTA, 20 mM HEPES, 100 mM K-aspartate, 40 mM KCl, 1 mM MgCl_2_, 2 mM maleic acid, 2 mM glutamic acid, 5 mM pyruvic acid, 0.5 mM KH_2_PO_4_, 5 mM MgATP, and pH adjusted to 7.2 with Trizma base) every 0.6 s (Nikon Eclipse Ti microscope) at 545 nm excitation using 20x objective to monitor mitochondrial Ca^2+^ dynamics. Confocal images were analyzed and quantified using ImageJ (National Institutes of Health, Bethesda, MD).

### Stable HL-1 Cell Lines

HL-1 cells (RRID:CVCL_0303) were a gift from William Claycomb (Louisiana State University) and were cultured as previously described (Claycomb et al., 1998). To knock down expression of the endogenous murine VDAC2 cells were lentivirally transduced with a construct expressing shRNA directed against murine VDAC2 (Min et al., 2012; Wilting et al., 2020) followed by selection using 3 μg/ml puromycin. This cell line was then again lentivirally transduced to overexpress zVDAC2, zVDAC2^E73Q^, zVDAC3, and zVDAC3^Q73E^ using respective constructs. Stable cell lines were established by selecting for nlsGFP expression by FACS sorting.

### SR-Mitochondria Ca^2+^ Transfer

Ca^2+^ transfer from the SR into mitochondria was measured as described previously (Schweitzer et al., 2017). In brief, HL-1 cardiomyocytes plated in a 96-well plate were loaded with 6 μM Rhod-2 AM (Life technologies) and 0.12% (w/v) Pluronic^®^ F-127 for 30 min at 37°C. After permeabilizing the cells with 100 mM digitonin in internal solution (in mM: 1 BAPTA, 20 HEPES, 100 L-Aspartic acid potassium salt, 40 KCl, 0.5 MgCl_2_, 2 maleic acid, 2 glutamic acid, 5 pyruvic acid, 0.5 KH_2_PO_4_, 5 MgATP, and 0.46 CaCl_2_; pH = 7.2 with KOH), cells were washed with internal solution before measurements. Rhod-2 fluorescence was monitored at excitation wavelength 540 ± 9 nm and emission wavelength 580 ± 20 nm with an Infinite^®^ 200 PRO multimode reader (Tecan, Maennedorf, Switzerland). After recording 30 s of baseline fluorescence, 10 mM caffeine was added to release Ca^2+^ from the SR.

### Statistical Analysis

Data are expressed as mean ± s.e.m. Statistical testing was carried out using student *t*-test unless otherwise specified. *Significance levels are expressed as *p < 0.05; **p < 0.01; ***p < 0.001; and NS not significant.

## Results

### Identification of Zebrafish VDAC Genes

**Figure 1:**
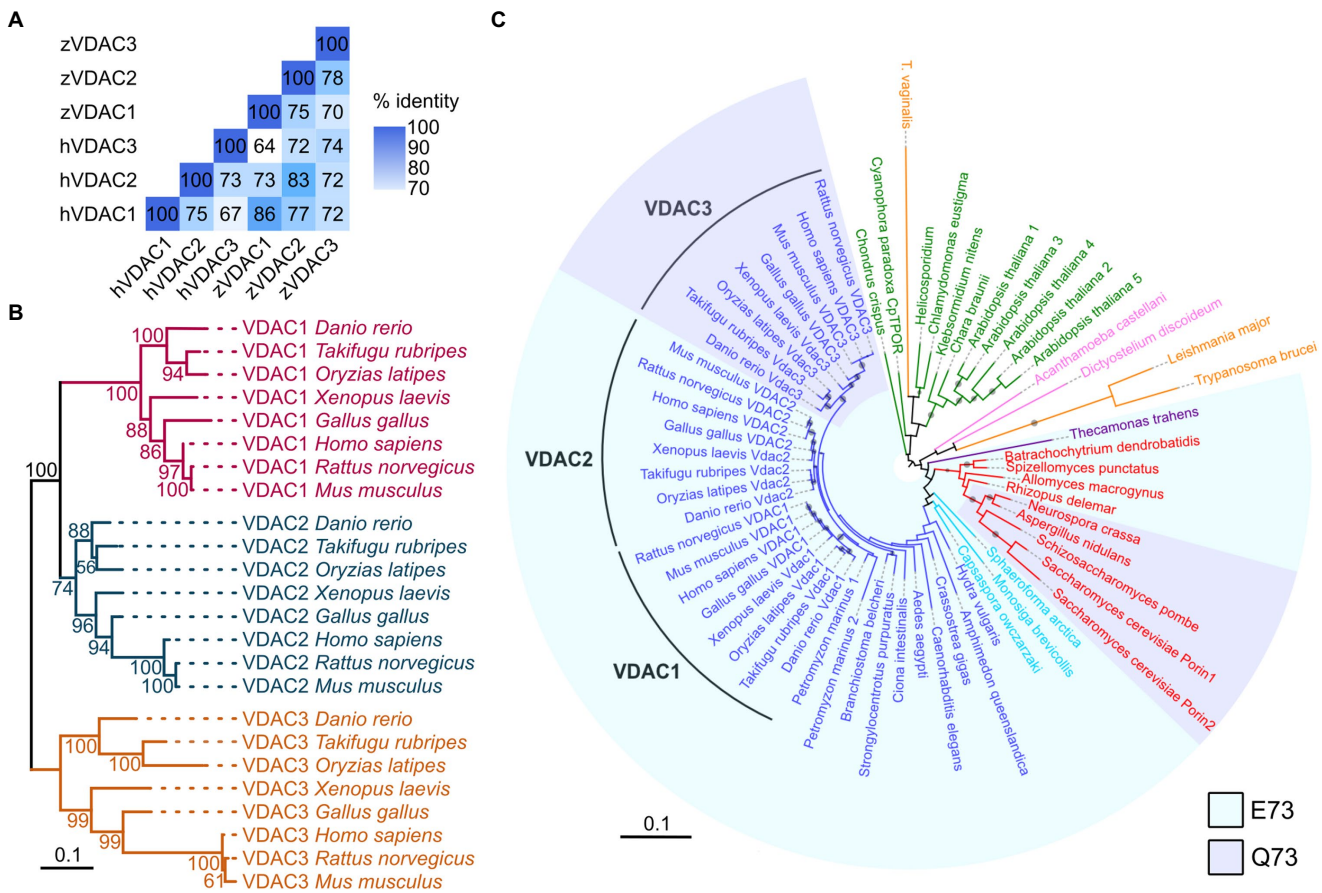
VDACs are highly conserved proteins. **(A)** Pairwise comparison of sequence identity of human (h) and zebrafish (z) VDAC protein sequences. **(B)** Maximum likelihood phylogenetic analysis of 24 vertebrate VDAC proteins. Branches are colored based on VDAC gene families (VDAC1: magenta, VDAC2: blue, and VDAC3: gold). Nodes are labeled with bootstrap values in units of percentage. The scale bar for branch lengths indicates the mean number of inferred substitutions per site. **(C)** Maximum likelihood phylogenetic analysis of 63 VDAC homologs from 41 species. Branches and labels are colored based on phylogenetic groupings of species: blue – animals, light blue – Holozoa (excluding animals), red – fungi, purple – Apusomonadida, orange – Excavata, pink – Amoebozoa, and green – Archaeplastida. Clades are shaded to indicate if proteins contain glutamate (E, light blue) or glutamine (Q, light purple) at amino acid position 73 (with respect to human VDAC1) or are unshaded if this region of the protein lacks high homology with human VDAC1. The size of circles on branches represents bootstrap values and is only displayed for values of 50% and greater. The scale bar for branch lengths indicates the mean number of inferred substitutions per site.

In vertebrates, the VDAC gene family consists of three paralogs: VDAC1, VDAC2, and VDAC3 (Naghdi and Hajnóczky, 2016). We examined the zebrafish genome and found three genes encoding VDAC proteins with high sequence identity to human VDAC1, VDAC2, and VDAC3 (Figure 1A). To investigate whether these three zebrafish proteins represent homologs of the mammalian VDACs and examine the evolutionary relationships among VDAC homologs, we constructed phylogenetic trees of VDAC amino acid sequences using a maximum likelihood approach. Consistent with the findings of previous studies (Young et al., 2007; Wojtkowska et al., 2012), vertebrate VDACs clustered in three distinct clades representing homologs of VDAC1, VDAC2, and VDAC3 (Figure 1B). Within each VDAC clade, zebrafish VDACs clustered tightly with the VDACs of other fish species (medaka and pufferfish). Importantly, one zebrafish VDAC protein was a member in each VDAC clade based on strong bootstrap support, indicating that zebrafish VDAC1, VDAC2, and VDAC3 are the bona fide homologs of their mammalian equivalents (Figure 1B).

Interestingly, while some plants and fungi possess multiple VDAC paralogs, only a single VDAC protein is present in the majority of the invertebrate animal species surveyed (Figure 1C). This suggests that the three VDAC proteins present in zebrafish likely arose from duplication events occurring in a common ancestor of teleost and tetrapod animals (amphibians, reptiles, birds, and mammals). The retention of the three VDAC paralogs throughout vertebrate evolution may indicate that they have acquired differing and crucial functional roles in mitochondrial biology.

### All Three VDAC Isoforms Are Expressed in the Hearts of Embryonic and Adult Zebrafish

**Figure 2:**
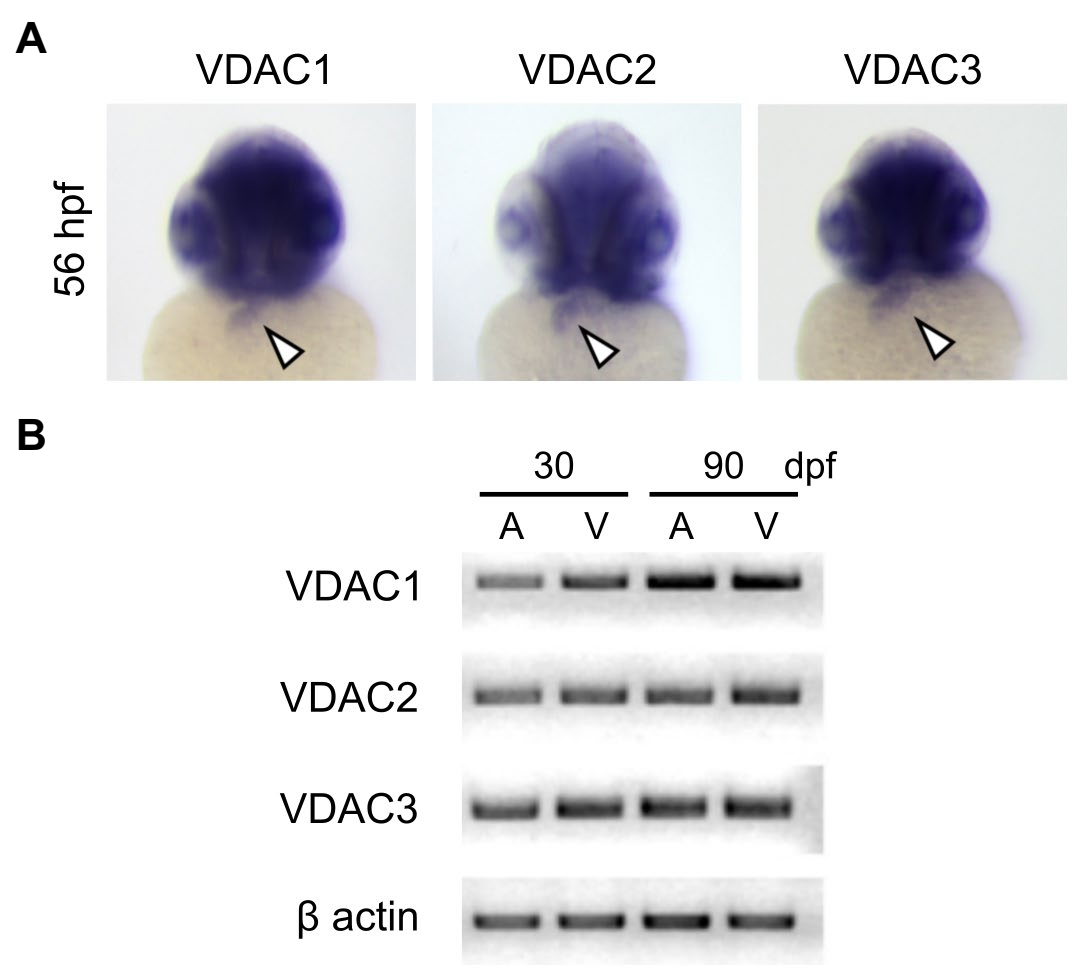
Expression of VDAC isoforms during zebrafish embryonic development. **(A)** Whole-mount *in situ* hybridization analysis using full-length riboprobes reveals that all VDAC isoforms are expressed in the embryonic heart (arrowheads). **(B)** RT-PCR analysis of *VDAC* mRNA from zebrafish hearts at 30 and 90 days post-fertilization (dpf). Expression of all VDAC isoforms is maintained to adulthood in both the atrium **(A)** and the ventricle (V).

We have previously shown that injecting wild-type VDAC2 mRNA in *tremblor/ncx1h* mutant embryos at the 1-cell stage allows *tremblor/ncx1h* mutant hearts to establish rhythmic Ca^2+^ transients and consequently preserves the integrity of myofibrils and maintains coordinated cardiac contractions (Shimizu et al., 2015, 2017). To investigate whether all three VDAC genes share this cardioprotective activity, we first examined if zebrafish VDAC1, 2, and 3 are expressed in the developing zebrafish heart. Whole-mount *in situ* hybridization revealed that all three VDACs are expressed in the embryonic heart (Figure 2A). Strong signals of VDAC transcripts are also detected in adult zebrafish hearts (both atrium and ventricle; Figure 2B), suggesting a role for VDAC proteins in the maintenance of cardiac physiology.

### Diverged Cardioprotective Effects Among VDAC Genes

**Figure 3:**
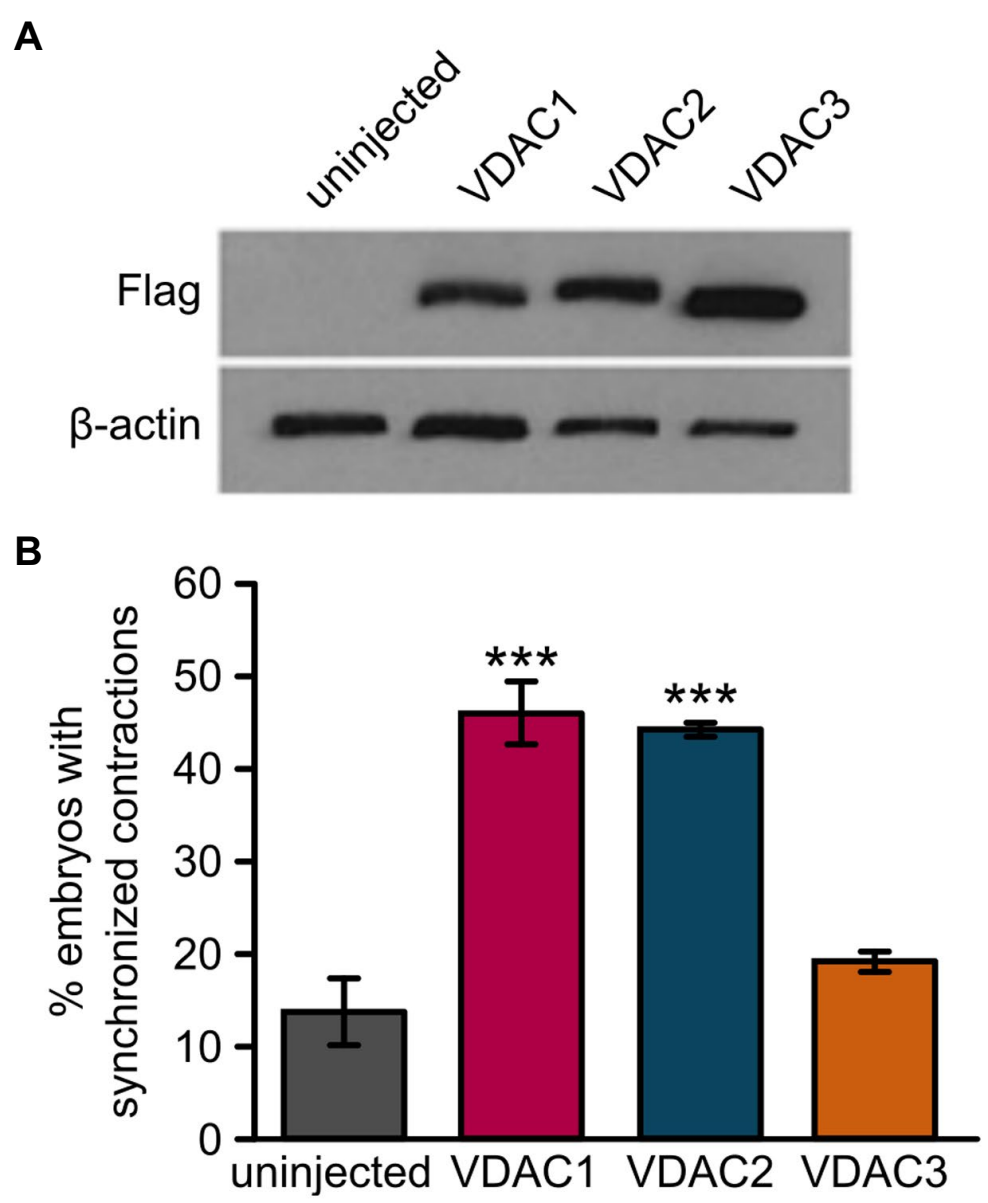
Overexpression of VDAC1 or VDAC2 but not VDAC3 restores rhythmic cardiac contractions in *tremblor/ncx1h* mutants. **(A)** Western blot with anti-FLAG antibody after SDS-PAGE with lysate from 32 hpf uninjected embryos or embryos injected with 25 pg. of C-terminally FLAG-tagged VDAC mRNA demonstrates that all VDAC mRNAs are expressed at comparable levels in embryos. β-actin was used as a loading control. **(B)** While injection of 25 pg. VDAC1 or VDAC2 mRNA significantly restored rhythmic cardiac contractions in 1-day-old *tremblor/ncx1h* mutant embryos (46.5 ± 3.4%, N = 3, *n* = 120, and 44.8 ± 0.4%, N = 3, *n* = 134, respectively, as opposed to 14 ± 3.5%, N = 3, *n* = 175 in uninjected siblings), injection of 25 pg. VDAC3 failed to recapitulate this effect (19.4 ± 1.1%, N = 3, *n* = 114). Overall rescue percentages represent the mean rescue percentage ± s.e.m. from N independent experiments, using a total of n embryos.

To evaluate the cardioprotective potential of VDAC1 and VDAC3, we injected FLAG-tagged VDAC1, 2, or 3 RNA into *tremblor/ncx1h* at the 1-cell stage (Figure 3). As expected, the majority of uninjected *tremblor/ncx1h* mutant hearts fibrillates where each individual cardiomyocytes contracts spontaneously but fails to coordinate with other cardiomyocytes within the same chamber to support a heartbeat (Langenbacher et al., 2005). However, a significantly higher number of FLAG-tagged VDAC2 RNA injected mutant embryos establish persistent and coordinated cardiac contractions as previous described (Shimizu et al., 2015, 2017), confirming a cardioprotective effect of VDAC2 against aberrant Ca^2+^ handling-induced arrhythmia. Interestingly, we observed that overexpression of VDAC1 and VDAC3 resulted in different effects on *tremblor/ncx1h* hearts. Similar to VDAC2, *tremblor/ncx1h* mutant embryos receiving VDAC1 mRNA established persistent cardiac contractions but the hearts of *tremblor/ncx1h* mutant embryos receiving VDAC3 RNA continued to fibrillate despite comparable expression levels as assessed by Western blot analysis against the flag epitope. These data demonstrate divergent cardioprotective effects among VDAC isoforms.

### Cardiomyocyte-Specific Expression of VDAC1 and VDAC2 Is Sufficient to Restore Cardiac Contractions in ncx1 Deficient Mutants

**Figure 4:**
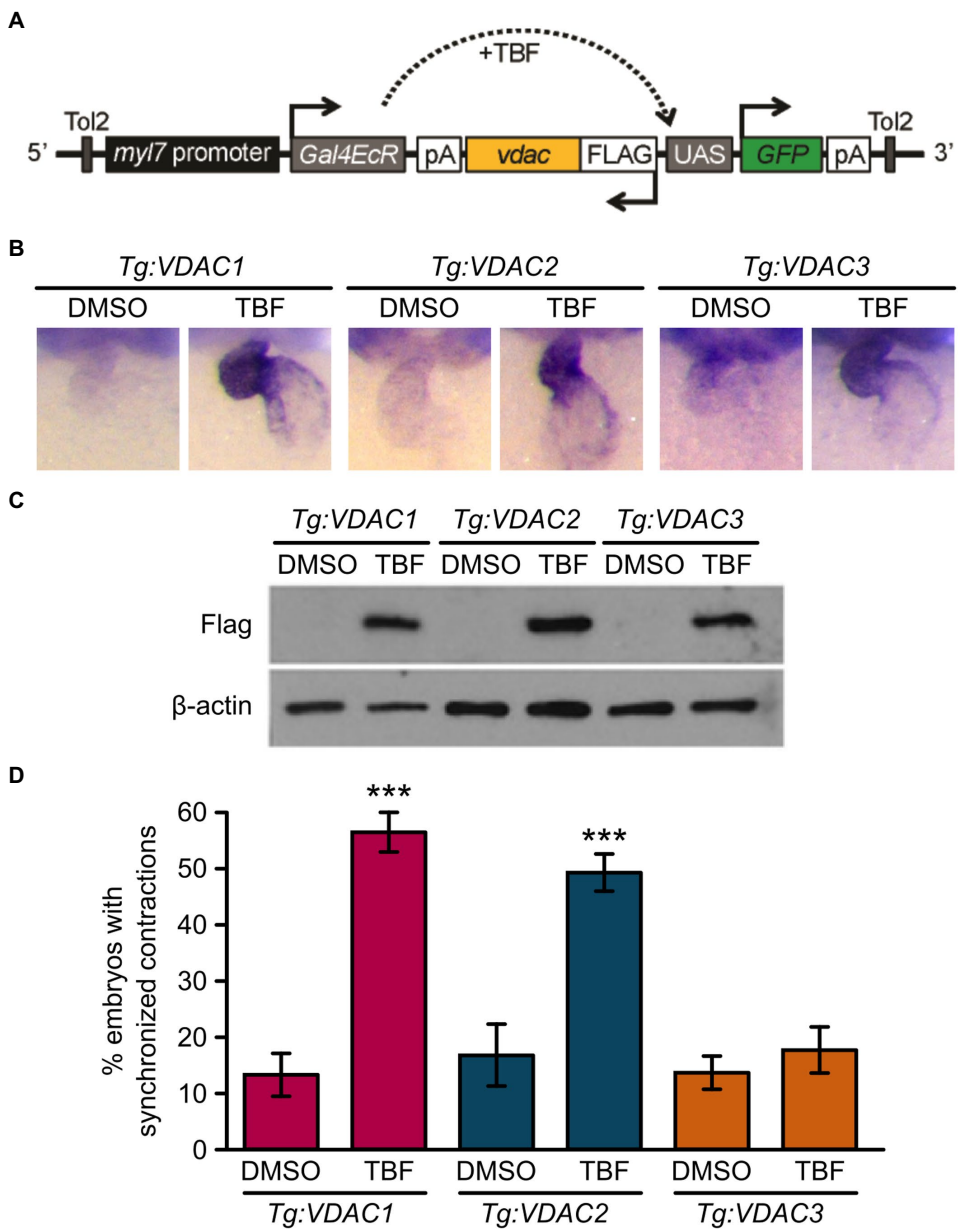
Induction of VDAC1 and VDAC2 but not VDAC3 expression restores rhythmic cardiac contraction in transgenic *tremblor/ncx1h* lines. **(A)** Schematic diagram of VDAC transgenic construct. The cardiomyocytes-specific promoter *myl7* drives Gal4-ecdysone receptor fusion protein (Gal4EcR), which becomes transcriptionally activated in response to tebufenozide (TBF), an ecdysone receptor agonist and binds to the upstream activating sequence (UAS), resulting in the simultaneous expression of both FLAG-tagged VDAC and EGFP. Transgenic lines were bred in the *tremblor/ncx1h* background. **(B)** Whole-mount *in situ* hybridization analysis demonstrating that VDAC expression is induced specifically in the heart upon TBF treatment in transgenic zebrafish (*Tg:VDAC*). Embryos were treated with either DMSO or TBF from 24 hpf until they were fixed for *in situ* hybridization at 48 hpf. **(C)** Western blotting of 32 hpf transgenic embryo lysate with an anti-FLAG antibody showing that VDAC protein expression is induced after embryos are treated with TBF. β-actin was used as a loading control. **(D)** While only 13.5 ± 3.6% of DMSO-treated *Tg:VDAC1; tremblor/ncx1h* embryos exhibit rhythmic cardiac contraction (N = 3, *n* = 233), 56.72 ± 3.5% of TBF-treated *Tg:VDAC1; tremblor/ncx1h* embryos established rhythmic contraction (N = 3, *n* = 238). Similarly, as opposed to only 16.8 ± 5.7% of DMSO-treated *Tg:VDAC2; tremblor/ncx1h* embryos (N = 3, *n* = 161), 49.6 ± 3.1% of TBF-treated *Tg:VDAC2; tremblor/ncx1h* embryos showed cardiac contraction (N = 3, *n* = 227). In contrast, the effect of TBF-induced overexpression is minimal in *Tg:VDAC3; tremblor/ncx1h* (13.7 ± 2.9%, N = 3, *n* = 283 in DMSO-treated embryos compared to 17.9 ± 4.0%, N = 3, *n* = 373 in TBF-treated embryos). Overall rescue percentages represent the mean rescue percentage ± s.e.m. from N independent experiments, using a total of n embryos.

RNA injection into newly fertilized eggs results in a global upregulation of VDAC proteins prior to the onset of Ca^2+^ handling defects in *tremblor/ncx1h* mutant embryos. This approach lacks cell type specificity and does not address the question of whether VDAC activation in cardiomyocytes alone is sufficient to suppress aberrant Ca^2+^ handling-induced cardiac dysfunction. To gain temporal- and cell type-specific resolution of the divergent effects of VDAC proteins on *ncx1* deficient hearts, we created transgenic lines *Tg:VDAC1, Tg:VDAC2, and Tg:VDAC3*, where the expression of FLAG-tagged VDAC isoforms as well as the GFP reporter is regulated by the cardiac-specific promoter *myl7* driven Gal4-ecdysone receptor fusion protein (Gal4-EcR; Figure 4A). We then crossed these transgenic fish into the *tremblor/ncx1h* mutant background, subjected the embryos to either vehicle (DMSO) or tebufenozide (TBF) treatment after 1 day of development, at which time the primitive heart has already formed and the *tremblor* mutant hearts are fibrillating (Langenbacher et al., 2005).

In the absence of TBF, Gal4-EcR is inactive and the transgenes are not expressed (Figures 4B,C; Lu et al., 2017). *In situ* hybridization analysis showed that TBF treatment effectively induced VDAC expression in the heart (Figure 4B). Western blotting further confirmed the induction of VDAC proteins by TBF treatment and detected comparable levels of induced VDAC proteins among three transgenic lines (Figure 4C). As expected, the majority of DMSO-treated transgenic *ncx1* deficient hearts exhibited fibrillation-like chaotic cardiac movement; coordinated and persistent cardiac contractions were observed in only approximately 14% of these embryos. Excitingly, about half of the hearts of *Tg:VDAC1;tremblor/ncx1h* and *Tg:VDAC2;* and *tremblor/ncx1h* embryos stopped fibrillating after TBF induction and began to contract. The hearts of *Tg:VDAC3; tremblor/ncx1h* embryos, on the other hand, continued to fibrillate after TBF treatment (Figure 4D). These findings parallel the divergent cardioprotective effects of VDAC1, 2, and 3 revealed by our global overexpression approach. In addition, because the induction of VDAC proteins in *tremblor/ncx1h* embryos occurs after the manifestation of cardiac fibrillation, our results demonstrate a cardiomyocyte autonomous suppressive effect for VDAC1 and VDAC2 on aberrant Ca^2+^ handling-induced cardiac dysfunction.

### The N-Terminal Domain of VDAC Proteins Is Responsible for Their Divergent Cardioprotective Effect

**Figure 5:**
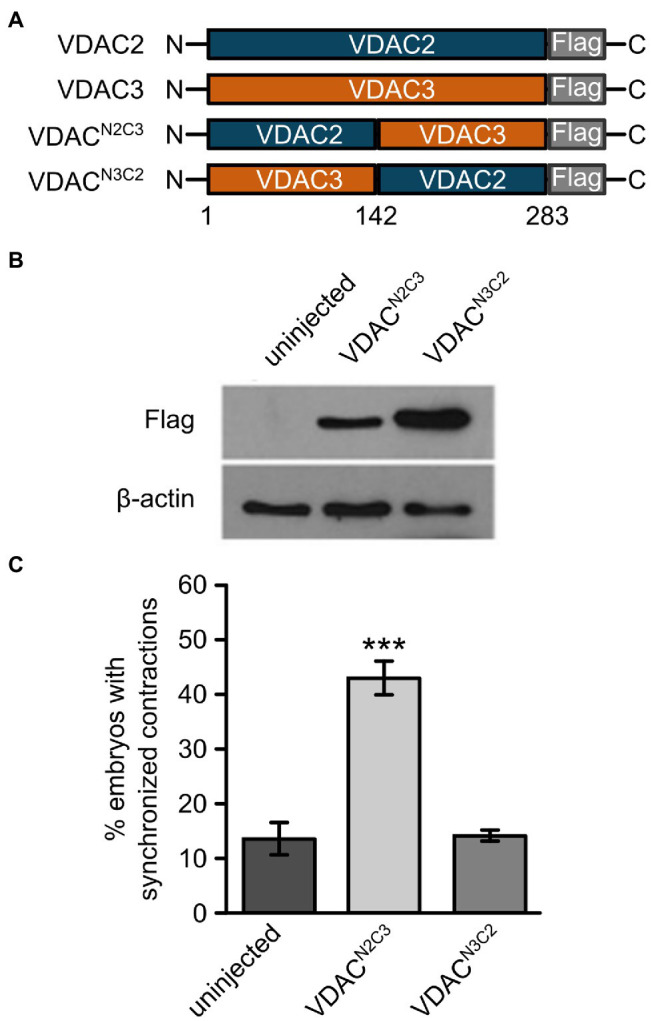
The N-terminal domain of VDAC2 contains critical elements for its cardioprotective activity. **(A)** Schematic representation of VDAC2/3 chimeric constructs. Reciprocal chimeric constructs were generated by swapping the N- and C-terminal halves of VDAC2 and VDAC3 and tagging a FLAG epitope at the C-terminal end. VDAC^N2C3^: The N-terminal half of VDAC2 (amino acids 1 to 142) was fused to the C-terminal half of VDAC3. VDAC^N3C2^: The N-terminal half of VDAC3 was fused to the C-terminal half of VDAC2. **(B)** Western blot with anti-FLAG antibody after SDS-PAGE with lysates from 30 hpf uninjected embryos or embryos injected with 60 pg. FLAG-tagged chimeric VDAC mRNA. β-actin was used as a loading control. **(C)** Injection of VDAC^N2C3^ mRNA restored synchronized cardiac contractions in 1-day-old *tremblor/ncx1h* embryos (43.4 ± 2.8%, N = 3, *n* = 168 compared to 13.7 ± 3.1%, N = 3, *n* = 156 in uninjected siblings), whereas injection of VDAC^N3C2^ mRNA fails to produce this effect (14.2 ± 1.2%, N = 3, *n* = 162). Overall rescue percentages represent the mean rescue percentage ± s.e.m. from N independent experiments, using a total of n embryos.

Mechanisms by which VDAC proteins regulate cardiac contraction remain elusive at the molecular level. The differing abilities of VDAC isoforms to restore of cardiac contractions in *tremblor/ncx1h* mutant hearts provide a reliable and robust platform to dissect critical domains for VDAC’s ability to suppress aberrant Ca^2+^ handling-induced arrhythmogenic effects in cardiomyocytes. We created FLAG-tagged chimeric constructs consisting of the N-terminal half of VDAC2 and the C-terminal half of VDAC3 (VDAC^N2C3^) and vice-versa (VDAC^N3C2^; Figure 5A). We then injected synthetic RNAs made from these chimeric constructs into *tremblor/ncx1h* embryos. While Western blots showed that both VDAC^N2C3^ and VDAC^N3C2^ were expressed at comparable levels (Figure 5B), only those *tremblor/ncx1*h mutant embryos receiving VDAC^N2C3^ mRNA manifested rhythmic cardiac contractions. The hearts of those mutant embryos receiving VDAC^N3C2^ RNA continued to fibrillate (Figure 5C). These findings indicate that the anti-arrhythmic activity of VDAC proteins is determined by amino acids located in their N-terminal half.

### E73 Is Evolutionary Conserved in VDAC1 and VDAC2

**Figure 6:**
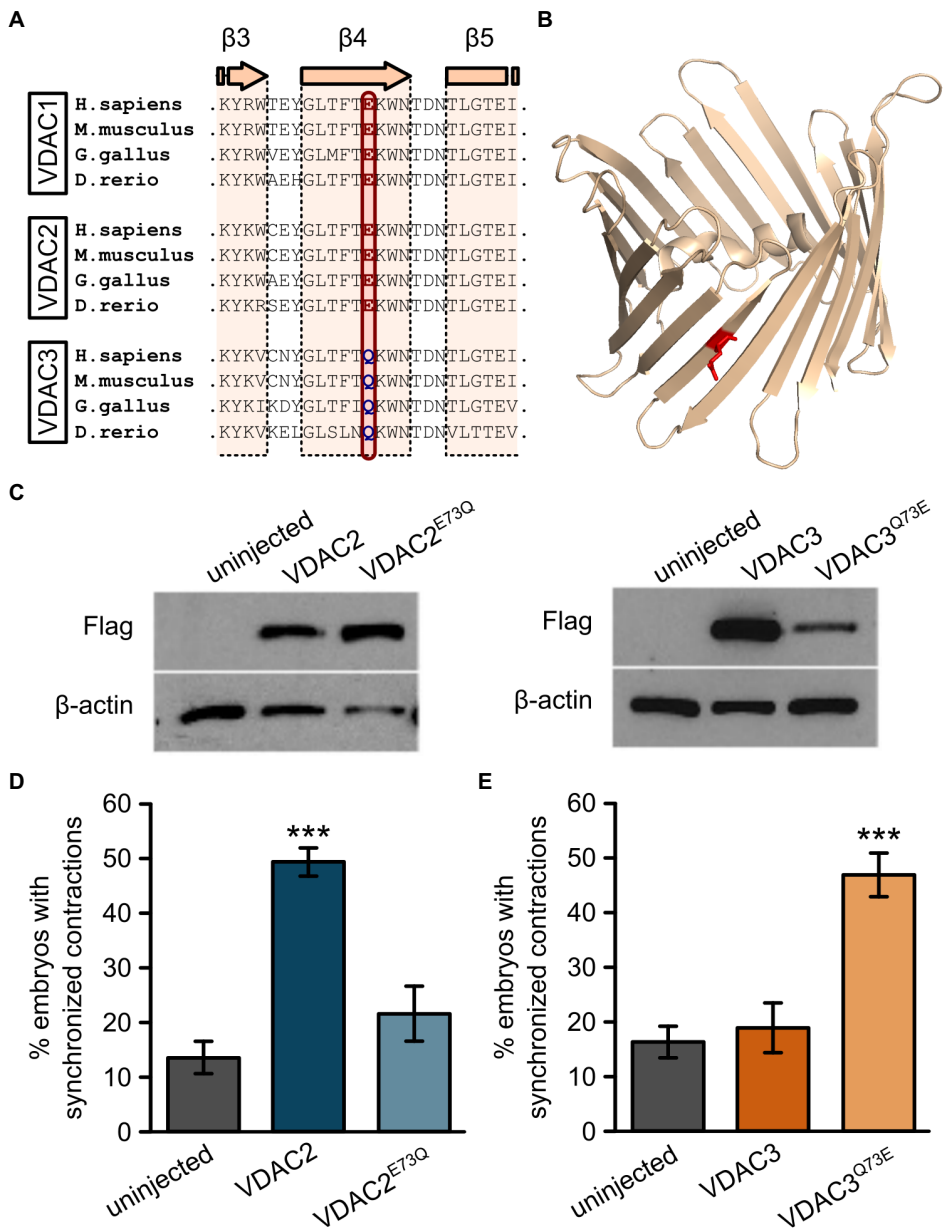
E73 is the critical amino acid residue that determines the ability of VDAC2 to suppress cardiac fibrillation in the *tremblor/ncx1h* mutant. **(A)** Alignment of protein sequences of VDAC1, 2, and 3 from different species. In all the species examined the position corresponding to zebrafish residue 73 is consistently occupied by a glutamate **(E)** in VDAC1 and VDAC2, whereas this position is occupied by glutamine (Q) in VDAC3. **(B)** Three-dimensional model of the VDAC2 protein (pdb: 4bum) showing the location of amino acid 73 in β-sheet 4 in red. (**C**) Western blot analysis of lysates from 30 hpf uninjected embryos or embryos injected with 25 pg. FLAG-tagged wild type and point mutant VDAC mRNA. β-actin was used as a loading control. (**D**) Mutation of E73 to Q in VDAC2 abrogates its ability to suppress cardiac fibrillation in *tremblor/ncx1h* mutants (49.7 ± 2.8%, N = 3, *n* = 144 with VDAC2 in contrast to 21.7 ± 5.1%, N = 3, *n* = 155 with VDAC2^E73Q^). (**E**) Vice-versa, by mutating Q73 to E, VDAC3 gained the ability to restore rhythmic cardiac contraction in *tre* mutants (19.0 ± 4.4%, N = 3, *n* = 182 with VDAC3 in contrast to 47.2 ± 4.3%, N = 3, *n* = 145 with VDAC3^Q73E^). Overall rescue percentages represent the mean rescue percentage ± s.e.m. from N independent experiments, using a total of n embryos.

While the N-terminal half of zebrafish VDAC1, 2, and 3 are highly similar (67–74% similarity at the amino acid level), the amino acid at position 73 (with respect to zebrafish VDAC2) differs between VDAC1 and 2, which exhibit anti-arrhythmic activity, and VDAC3, which does not (Figures 6A,B). Residue 73 is located in β-sheet 4 of the channel facing the outside of the barrel (Figure 6B) and a glutamate is present at this position in zebrafish VDAC2 (E73), while a glutamine is present at the corresponding position in zebrafish VDAC3 (Q73). E73 is highly conserved among animals; it is present in all animal VDAC proteins we examined excluding the VDAC3 family (Figure 1C).

Interestingly, E73 also appears in some fungi and in several protists (including choanoflagellates) that are believed to be closely related to animals (Figure 1C), suggesting an early evolutionary origin and subsequent conservation of this residue. While E73 is present in early evolutionary branches of fungi (Blastocladiomycota, Chytridiomycota, Chytridiomycota, and Mucoromycota), Q73 is instead present in fungi from the subkingdom Dikarya. Therefore, Q73 appears to have evolved twice, once in animals (VDAC3) and once in a subset of fungi (Dikarya). Intriguingly, like E73, Q73 also appears to be highly conserved within the taxa that it evolved (Figure 1C). Together, the simultaneous conservation of E73 and Q73 suggests that these two residues may impart significant functional differences to the VDAC protein that in turn created an evolutionary pressure for their retention.

Given that upregulation of mitochondrial Ca^2+^ uptake restores cardiac contraction in *tremblor/ncx1h* mutant embryos (Shimizu et al., 2015) and that E73 was previously reported to have an essential role in Ca^2+^ handling (Israelson et al., 2007; Peng et al., 2020), we suspected that E73 may be a critical residue that determines the isoform-specific rescue effect of VDAC genes on *tremblor/ncx1h*’s arrhythmia phenotype. To investigate this possibility, we created two point mutants: VDAC2^E73Q^ by replacing E73 in VDAC2 with a glutamine and VDAC3^Q73E^ by substituting Q73 of VDAC3 with a glutamate. We then injected VDAC2^E73Q^ and VDAC3^Q73E^ RNA into *tremblor/ncx1h* mutant embryos. Western blots showed that both VDAC2^E73Q^ and VDAC3^Q73E^ are stably expressed in embryos (Figure 6C). Interestingly, opposite to wild-type VDAC2 and VDAC3 overexpression effects, the hearts that received VDAC2^E73Q^ RNA continued to fibrillate, whereas VDAC3^Q73E^ overexpression suppressed cardiac fibrillation and restored rhythmic cardiac contraction in *tremblor/ncx1h* mutant embryos (Figures 6D,E).

### E73 Controls VDAC-Dependent Mitochondrial Ca^2+^ Uptake

**Figure 7:**
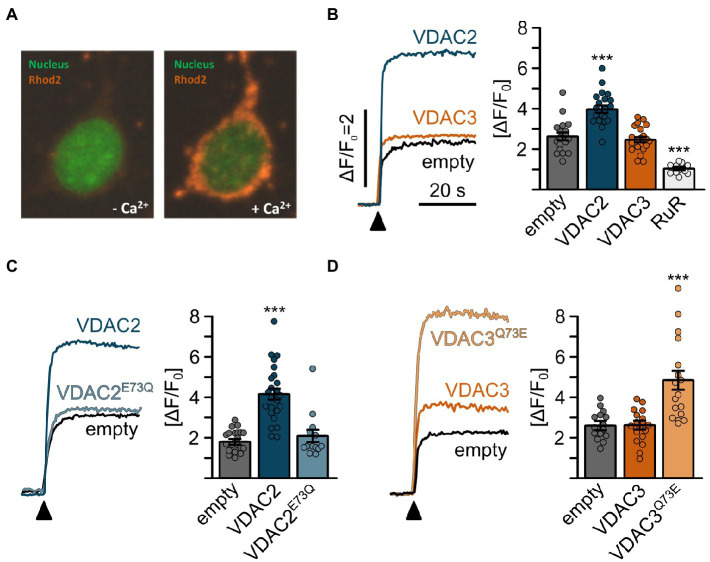
Mitochondrial Ca^2+^ uptake in HeLa cells is promoted by E73. **(A)** Representative confocal images of permeabilized HeLa cells loaded with Rhod2 after transiently transfection with VDAC expression constructs. Mitochondrial Rhod2 fluorescence is minimal at the basal state (left). Upon addition of Ca^2+^, the Rhod2 fluorescence rapidly concentrates in mitochondria (right). **(B-D)** Mitochondrial Ca^2+^ uptake assays of HeLa cells transiently transfected with VDAC constructs; **(B)** mitochondrial Ca^2+^ uptake was observed in empty vector transfected control cells (empty, ∆F/F_0_ = 2.64 ± 0.82, *n* = 18), which was significantly enhanced by overexpression of VDAC2 (∆F/F_0_ = 3.99 ± 0.80, *n* = 22) but not VDAC3 (∆F/F_0_ = 2.47 ± 0.61, *n* = 22) and significantly inhibited by ruthenium red (∆F/F_0_ = 1.06 ± 0.23, *n* = 12). **(C)** While wild-type VDAC2 enhanced mitochondrial Ca^2+^ uptake (∆F/F_0_ = 1.83 ± 0.52 for empty cells vs. ∆F/F_0_ = 4.18 ± 1.4 for VDAC2, *n* = 22 and 24, respectively), VDAC2^E73Q^ failed to induce this effect (∆F/F_0_ = 2.09 ± 1.15, *n* = 13). **(D)** Conversely, wild-type VDAC3 did not induce a significant increase in mitochondrial Ca^2+^ uptake (∆F/F_0_ = 2.63 ± 0.70 for empty cells vs. ∆F/F_0_ = 2.59 ± 0.81 for VDAC3, *n* = 15 and 18, respectively); however, VDAC3^Q73E^ significantly increased mitochondrial Ca^2+^ uptake to ∆F/F_0_ = 4.85 ± 1.99 (*n* = 18).

VDAC2 activation suppresses cardiac fibrillation in *tremblor/ncx1h* mutants by buffering excess Ca^2+^ into the mitochondria (Shimizu et al., 2015). To test whether VDAC’s differential cardioprotective activities on *tremblor/nxc1h* mutant hearts are rooted in their differential Ca^2+^ transporting activity, we transfected HeLa cells with VDAC isoforms and measured mitochondrial Ca^2+^ uptake using Rhod-2 (Figure 7A). Cells transfected with VDAC2 showed increased mitochondrial Ca^2+^ levels compared to empty vector transfected control cells upon stimulation, whereas no significant differences in mitochondrial Ca^2+^ uptake between control and VDAC3 transfected cells were observed (Figure 7B). Given the previously observed correlation between position 73 and the cardioprotective activity of VDAC2 and VDAC3, we next examined whether E73 might determine the differential mitochondrial Ca^2+^ uptake activities between VDAC isoforms. Indeed, wild-type VDAC2 enhanced mitochondrial Ca^2+^ uptake, while VDAC2^E73Q^ showed a mitochondrial Ca^2+^ uptake profile comparable to control cells. In contrast, VDAC3 did not enhance mitochondrial Ca^2+^ uptake above control, while introduction of E73 into VDAC3 (VDAC3^Q73E^) significantly enhanced mitochondrial Ca^2+^ uptake, demonstrating that E73 is a key determinant of VDAC2’s mitochondrial Ca^2+^ uptake activity (Figures 7B–D).

### E73 Critically Regulates VDAC2-Mediated Transfer of Ca^2+^ From the SR Into Mitochondria

**Figure 8:**
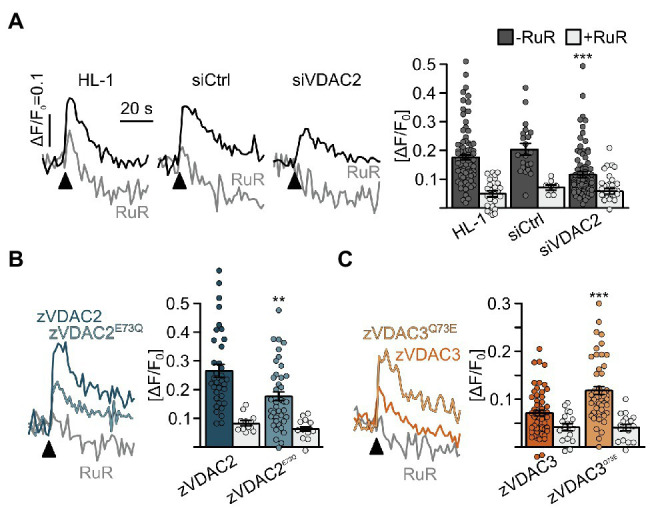
Restoration of SR-mitochondria Ca^2+^ transfer in permeabilized HL-1 cardiomyocytes. (**A**) Knockdown of the endogenous mVDAC2 in HL-1 cells significantly reduced SR-mitochondria Ca^2+^ transfer after addition of caffeine (arrowhead) from ΔF/F_0_ = 0.18 ± 0.01 (*n* = 84) to 0.12 ± 0.01 (*n* = 83) while a scrambled siRNA control did not affect Ca^2+^ transfer (0.20 ± 0.02, *n* = 21). (**B**) Wild-type zVDAC2 restored mitochondrial Ca^2+^ uptake to 0.27 ± 0.02 (*n* = 36), well above mVDAC2 knockdown cells. Overexpression of zVDAC2^E73Q^ restored mitochondrial Ca^2+^ uptake to only 0.18 ± 0.02 (*n* = 39), which is significantly lower compared to wild-type zVDAC2. (**C**) Conversely, zVDAC3 did not restore mitochondrial Ca^2+^ uptake in mVDAC2 knockdown cells (0.12 ± 0.01, *n* = 54), while VDAC3^Q73E^ restored mitochondrial Ca^2+^ uptake to ΔF/F_0_ = 0.20 ± 0.02, (*n* = 50). Arrowheads indicate injection of 10 mM caffeine. Statistical analysis was performed using Kruskal-Wallis test with Dunn’s *post-hoc* test.

Considerable differences in mitochondrial Ca^2+^ handling have been observed between excitable and non-excitable cells. In particular, in cardiomyocytes, VDAC2 was suggested to interact with RyR2, the major Ca^2+^ release channel on the SR, and thereby facilitate Ca^2+^ transfer from the SR to mitochondria (Subedi et al., 2011; Min et al., 2012). We therefore examined whether E73 regulates VDAC2’s ability to mediate the transfer of Ca^2+^ from the SR into mitochondria in cultured HL-1 cardiomyocytes (Claycomb et al., 1998; Schweitzer et al., 2017; Wilting et al., 2020). Stable knockdown of the endogenously expressed murine VDAC2 by siRNA (Subedi et al., 2011; Min et al., 2012; Wilting et al., 2020) significantly reduced uptake of Ca^2+^ released from the SR by a caffeine pulse into mitochondria, while this uptake was comparable to native HL-1 cells when a scrambled control shRNA was used (Figure 8). We subsequently overexpressed our VDAC constructs, VDAC2, VDAC2^E73Q^, VDAC3, and VDAC3^Q73E^ in mVDAC2 knockdown cells and evaluated their abilities to restore SR-mitochondria Ca^2+^ transfer. VDAC2 restored mitochondrial Ca^2+^ uptake to levels above baseline consistent with the idea of VDAC2 as the main mediator of SR-mitochondria Ca^2+^ transfer. Strikingly, replacing E73 of VDAC2 with a glutamine (VDAC2^E73Q^) abrogated its ability to promote SR-mitochondria Ca^2+^ transfer (Figure 8B). On the other hand, while VDAC3 failed to restore SR-mitochondria Ca^2+^ transfer, introduction of E73 in VDAC3^Q73E^ restored SR-mitochondria Ca^2+^ transfer to control levels (Figure 8C). These data indicate that VDAC-mediated SR-mitochondria Ca^2+^ transfer is dependent on the presence of E73 in cardiomyocytes.

## Discussion

Despite structural and functional similarities, divergent biological functions have been noted among VDAC isoforms. Accumulating evidence indicates that VDAC2 is a critical gatekeeper for mitochondrial Ca^2+^ entry in physiology and in disease within the heart (Min et al., 2012; Shimizu et al., 2015, 2017; Schweitzer et al., 2017; Wilting et al., 2020; Sander et al., 2021). In this study, we used zebrafish *tremblor/ncx1h* mutants as an *in vivo* platform to assess the ability of VDAC1 and VDAC3 to regulate cardiac rhythmicity through mitochondrial Ca^2+^ uptake. We demonstrate that like VDAC2, VDAC1 is capable of transporting Ca^2+^ across the outer mitochondrial membrane. When overexpressed, VDAC1 effectively restores rhythmic cardiac contractions in *ncx1* deficient hearts. VDAC3, on the contrary, cannot facilitate efficient Ca^2+^ transport across the mitochondrial membrane, demonstrating divergent roles for these isoforms in Ca^2+^ trafficking. Structure-function analysis further revealed that the evolutionarily conserved E73/Q73 confers this divergent VDAC isoform-dependent calcium trafficking function.

Across all vertebrate species, a glutamate occupies position 73 in VDAC1 and VDAC2 (E73), while a glutamine is present at this position in VDAC3 (Q73). Using multiple model systems, we demonstrate that only isoforms with E73 are able to promote mitochondrial Ca^2+^ uptake and to regulate cardiac rhythmicity. While VDAC3 did not mediate mitochondrial Ca^2+^ uptake in its wild-type form, replacing Q73 with a glutamate allows VDAC3 to facilitate SR-mitochondrial Ca^2+^ transfer, enhance mitochondrial Ca^2+^ uptake, and suppress cardiac arrhythmia. These data are consistent with the established notion that E73 is an important regulator of VDAC function. However, the precise mechanism by which E73 influences Ca^2+^ trafficking remains elusive. Our data suggest that E73 confers cardiac protection by facilitating mitochondrial Ca^2+^ uptake. In line with these findings, E73 has been suggested to be involved in the Ca^2+^ transport activity of VDAC (Gincel et al., 2001; Ge et al., 2016; Peng et al., 2020) and is also the binding site for VDAC blockers like ruthenium red and its derivatives, which compete with Ca^2+^ for binding to this site (Israelson et al., 2007). Also supporting the notion that E73 can facilitate the inter-organellar transport of Ca^2+^ is the recent finding that it influences mitochondrial uptake of Ca^2+^ from lysosomes (Peng et al., 2020). Given the location of E73 on the outside of the fourth repeat of the VDAC barrel facing the lipid membrane (Ujwal et al., 2008; Schredelseker et al., 2014), it is unlikely that E73 serves as a direct binding site for Ca^2+^ ions, but instead may indirectly promote the uptake of Ca^2+^ by VDAC. Indeed, E73 alone was unable to modify voltage gating of purified VDAC1 channels (Queralt-Martín et al., 2019). An indirect effect is further supported by the findings that the positive charge of E73 can induce a thinning of the local plasma membrane (Villinger et al., 2010) and that E73 promotes binding of VDAC protein partners like hexokinase (Abu-Hamad et al., 2008) and mediates VDAC dimerization (Bergdoll et al., 2018). Though our data demonstrate an essential role for E73 in mitochondrial Ca^2+^ uptake, these reports clearly highlight the need for further biochemical and structural studies to fully elucidate the molecular mechanisms of E73’s involvement in VDAC-mediated Ca^2+^ transit.

In cardiomyocytes, VDAC2 interacts with the ryanodine receptor to shape intracellular Ca^2+^ signals (Subedi et al., 2011; Min et al., 2012). Knockout of VDAC2 alone is sufficient to attenuate SR-mitochondria Ca^2+^ transfer in cardiomyocytes and cardiac-specific elimination of VDAC2 was reported to result in early cardiac dysfunction (Raghavan et al., 2012). While our study suggests that both VDAC1 and VDAC2 are capable of mediating mitochondrial Ca^2+^ uptake in cardiomyocytes, VDAC2 appears to be more relevant to mammalian cardiac biology. VDAC1 is known to couple with the IP3-receptor to mediate mitochondrial Ca^2+^ uptake in non-excitable cells. However, inactivation of glycogen synthase kinase-3*β* was shown to reduce coupling of the IP3-receptor to VDAC1 in cardiomyocytes and to reduce mitochondrial Ca^2+^ uptake during cardiac ischemia-reperfusion (Gomez et al., 2016). It would thus be interesting to explore differential interaction of VDAC1 and VDAC2 with the ryanodine receptor or other Ca^2+^ release channels on various organelles to determine if VDAC2 plays a more acute and VDAC1 plays a more subtle role in healthy cardiomyocytes.

The presence of the highly conserved VDAC3 family, which lacks E73, in vertebrates suggests that this difference may impart crucial functional properties to VDAC3. Whether the VDAC proteins in vertebrates have evolved subtype-specific distinct functions, with each one assuming part of the role played by the single VDAC protein in basal metazoans, or if they have acquired novel functions are still an open question that could be examined to better understand the role of mitochondria in the evolution of the highly demanding vertebrate heart muscle. Whether the E73/Q73 residues identified in fungi by sequence homology are functionally and structurally homologous to zebrafish VDAC, E73/Q73 has not been studied. It would be interesting to examine whether E73 containing and/or Q73 containing fungal VDACs could substitute for the activity of VDAC2 in zebrafish embryos or mammalian cells, and whether these proteins exhibit differing calcium permeability properties. Most species of fungi examined have only a single VDAC protein, with the notable exception of *S. cerevisiae*. Q73 is present in Dikarya, a subkingdom of Fungi, while E73 is present in all other fungal species surveyed. It is intriguing to note that similar to the evolution of vertebrate VDACs, fungal E73 and Q73 are conserved within their respective clades, suggesting that differences at this residue may be functionally relevant. If indeed Q73 reduces the calcium permeability of fungal VDACs, this would suggest that a reduced requirement for mitochondrial calcium uptake or alternative routes of mitochondrial calcium uptake may have evolved in Dikarya. Alternatively, structural differences between animal and fungal VDACs may render fungal E73 less crucial for calcium uptake.

Together, our data demonstrate that the evolutionarily conserved E73 residue mediates the divergent anti-arrhythmic effects of VDAC isoforms by modulating mitochondrial Ca^2+^ uptake in cardiomyocytes. These findings underscore the important role of mitochondria in cellular Ca^2+^ dynamics and suggest that further exploring VDAC1- and VDAC2-mediated mitochondrial Ca^2+^ uptake may reveal novel regulatory mechanisms for normal cardiac physiology and heart disease.

## Data Availability Statement

The datasets presented in this study can be found in online repositories. The names of the repository/repositories and accession number(s) can be found in the article/Supplementary Material.

## Ethics Statement

The animal study was reviewed and approved by University of California Los Angeles Animal Care and Use Committee.

## Author Contributions

HS, SH, AL, LC, JH, KW, FW, and JS performed the experiments. JS and J-NC conceptualized the study. HS, JS, TG, and J-NC acquired the funding. HS, AL, LC, JS, and J-NC wrote the manuscript. All authors commented on the manuscript.

## Conflict of Interest

The authors declare that the research was conducted in the absence of any commercial or financial relationships that could be construed as a potential conflict of interest.

## Publisher’s Note

All claims expressed in this article are solely those of the authors and do not necessarily represent those of their affiliated organizations, or those of the publisher, the editors and the reviewers. Any product that may be evaluated in this article, or claim that may be made by its manufacturer, is not guaranteed or endorsed by the publisher.

## Footnotes

^1^ https://www.ncbi.nlm.nih.gov/
