# Supplementary material for: Glutamate 73 Promotes Anti-arrhythmic Effects of Voltage-Dependent Anion Channel Through Regulation of Mitochondrial Ca2+ Uptake
Source: Front Physiol. 2021 Aug 18;12:724828. doi: 10.3389/fphys.2021.724828 (PMC8416314; doi:10.3389/fphys.2021.724828)
Supplement: Supplementary file 1 [file Data_Sheet_1.PDF]

SUPPLEMENTARY INFORMATION FOR

Glutamate 73 promotes anti-arrhythmic effects of VDAC through regulation of SR-mitochondria Ca^{2+} transfer

Supplementary File 1. Species origins and NCBI accession IDs for protein sequences used in phylogenetic analyses. Column A contains the label given to the sequence in the phylogenetic trees. Column B contains the GenBank or NCBI Reference ID for the sequence used in the phylogenetic analysis. Column C contains the common name of the species from which the protein sequence is derived.

See Table 1.

Supplementary File 2. Primer sequences.

Experiment	Target gene	Sequence
RT-PCR	*vdac1*	F: 5'- CACGTCTCTCCTCTCCACTGTCG -3'
		R: 5'- CCGTCCCGTTGATGTCGTAGTCCAC -3'
	vdac2	F: 5'- CTCAGCGCTGGGAATAACTTCAGCAG -3'
		R: 5'- CTGTGTTTGGTGAGAAGGTTGTGTCAAAGG -3'
	vdac3	F: 5'- GTTCTCCTTCAGTGTGCCCTCTC -3'
		R: 5'- CCAGTGTTGGGAACAAATGAAGTGTCC -3'
Quantitative RT-PCR	*vdac1*	F: 5'- CACGTCTCTCCTCTCCACTGTCG -3'
		R: 5'- CATATCCTTTGGTGAAGATGTCTCTGGCAG -3'
	vdac2	F: 5'- CTCAGCGCTGGGAATAACTTCAGCAG -3'
		R: 5'- CGAGCTTCACCATACCGAATCCATATCC -3'
	vdac3	F: 5'- GGAGTGGGATACACACAGAGCCTC -3'
		R: 5'- CCTGTCAGTCGTTAAACCTCCAGC -3'
